# Ancient ocean coastal deposits imaged on Mars

**DOI:** 10.1073/pnas.2422213122

**Published:** 2025-02-24

**Authors:** Jianhui Li, Hai Liu, Xu Meng, Diwen Duan, Haijing Lu, Jinhai Zhang, Fengshou Zhang, Derek Elsworth, Benjamin T. Cardenas, Michael Manga, Bin Zhou, Guangyou Fang

**Affiliations:** ^a^School of Civil Engineering and Transportation, Guangzhou University, Guangzhou 510006, China; ^b^Institute of Geology and Geophysics, Chinese Academy of Sciences, Beijing 100029, China; ^c^Department of Geotechnical Engineering, Tongji University, Shanghai 200092, China; ^d^Department of Energy and Mineral Engineering, The Pennsylvania State University, University Park, PA 16802; ^e^Department of Geosciences, The Pennsylvania State University, University Park, PA 16802; ^f^Department of Earth and Planetary Science, University of California, Berkeley, CA 94720; ^g^Aerospace Information Research Institute, Chinese Academy of Sciences, Beijing 100094, China

**Keywords:** Mars, ancient ocean, Zhurong rover, sedimentary deposits, ground penetrating radar

## Abstract

The northern lowlands of early Mars could have contained a significant quantity of liquid water. However, the ocean hypothesis remains controversial due to the lack of conclusive evidence from the Martian subsurface. We use data from the Zhurong Rover Penetrating Radar on the southern Utopia Planitia to identify subsurface dipping reflectors indicative of an ancient prograding shoreline. The reflectors dip unidirectionally with inclinations in the range 6° to 20° and are imaged to a thickness of 10 to 35 m along an uninterrupted 1.3 km northward shoreline-perpendicular traverse. The consistent dip inclinations, absence of dissection by fluvial channels along the extended traverse, and low permittivity of the sediments are consistent with terrestrial coastal deposits—and discount fluvial, aeolian, or magmatic origins favored elsewhere on Mars. The structure, thickness, and length of the section support voluminous supply of onshore sediments into a large body of water, rather than a merely localized and short-lived melt event. Our findings not only provide support for the existence of an ancient Martian ocean in the northern plains but also offer crucial insights into the evolution of the ancient Martian environment.

## Significance

Various observations suggest that large amounts of liquid water once existed on the Martian surface, however, the nature and fate of this water are uncertain. Through radar data gathered by the Zhurong Rover, we identify extensive dipping deposits in the subsurface of southern Utopia Planitia. These deposits have structures similar to those of Earth’s coastal sediments. This finding implies the past existence of a large water body, supporting the hypothesis of a past ocean in the northern plains of Mars.

**Fig. 1.**
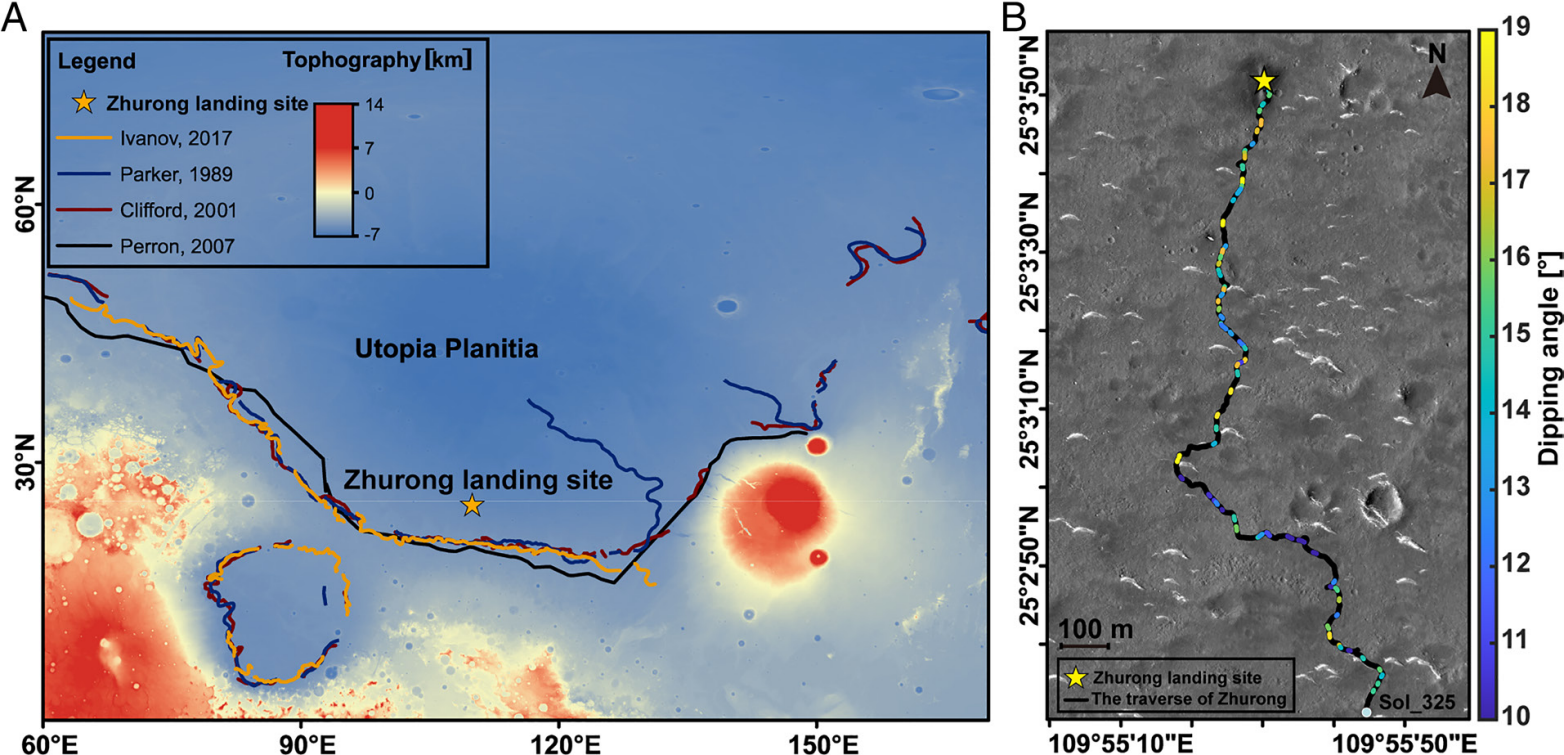
Zhurong rover landing site and proposed shorelines in Utopia Planitia. (*A*) Map of Utopia Planitia, showing the landing site of the Zhurong rover and four proposed ancient shorelines (9, 11, 22, 23). The Zhurong landing site is ~280 km north of and ~500 m lower in elevation than the northern hypothesized shorelines (6). (*B*) HiRISE image (ESP_073225_2055) with the traverse of the Zhurong rover from Sol 11 to Sol 325. The color marked along the traverse indicates the dip angles identified by the RoPeR data. Image credit of HiRISE: NASA/JPL/University of Arizona.

Mars expresses Earth-like geological features, seasonal cycles, and day–night rhythm making it a unique analog to the evolution of the Earth and informing the search for extraterrestrial life. Although the surface of present-day Mars is cold and dry (1), geological features such as valley networks (2), open- and closed-basin lakes (3), deltas (4), alluvial fans (5), pitted-cones (6), and sedimentary rocks (7, 8) support the prior existence of vast amounts of liquid water. Possible paleoshorelines identified from orbital images might also map the extent of ancient oceans in the northern lowlands that would have covered a third of the Martian surface (9). While the mapped boundaries occupy multiple elevations, challenging the ocean hypothesis (10), true polar wander (11), and loading from the formation of Tharsis (12) might explain some of the long-wavelength deformation of old shorelines. Reconciling ages and large unexplained topographic variations, however, continues to cast doubt on whether reported features are in fact paleoshorelines (13). The Martian northern lowland surface has experienced long-term weathering, aeolian deposition, impact resurfacing, and other geomorphologic adjustments (14), which potentially distort or mask the observational record of an ancient ocean. Evidence from sedimentary deposits (8, 15, 16), particularly direct evidence from in situ investigations can potentially reveal deposits from an ocean. China’s first Mars rover “Zhurong” successfully landed on the southern Utopia Planitia (109.925°E, 25.066°N) and began its exploration toward the proposed shorelines on 15 May 2021 (Fig. 1) (17, 18). Before its forced hibernation on May 18, 2022, the rover had traveled 1,921 m and collected a wealth of scientific data with its many payloads (19). Of greatest relevance in searching for subsurface structures that are a legacy of an ocean is the Rover Penetrating Radar (RoPeR) dual-channel ground penetrating radar (GPR) system (20 and *SI Appendix*, Fig. S1). It operates at low frequency for deep penetration (15 to 95 MHz) and high frequency for high resolution (0.45 to 2.15 GHz). The primary objectives of RoPeR are to define subsurface structures and the possible presence of water ice beneath the landing region (19–21).

The landing site is within a late Hesperian lowland unit composed of Vastitas Borealis Formation (VBF) materials. The VBF is widely distributed across the Martian northern lowlands and is therefore interpreted as possible sedimentary deposits. However, it is still unclear whether the VBF originates from fluvial, lacustrine, or marine processes. The Zhurong rover has already contributed significant findings to resolve surface characteristics (21, 24–26), subsurface structures (20) and dielectric properties of Martian soil (27), including those of marine sedimentary rocks ejected by meteorite impacts at the landing site (7). These studies demonstrate that the landing area underwent various water-related processes and also raise the intriguing possibility that sedimentary deposits from an ancient ocean might lie beneath the surface and be potentially detectable by RoPeR. We use the low-frequency subsurface radar reflectors recorded by RoPeR to image shallow structures beneath the regolith and provide subsurface constraints for the presence and form of a past-ocean shoreline. We identify dipping reflectors that provide strong subsurface evidence for the presence of an ancient ocean on Mars during the Late Hesperian period.

## Results

The Zhurong rover landed on the surface of southern Utopia Planitia, within reach of previously proposed shorelines. Previous studies proposed that sedimentary deposits in the landing area had been reworked by water (7, 20). We reconstruct sedimentary structures from the radar data and compare geometries with analogs in terrestrial coastal environments.

**Fig. 2.**
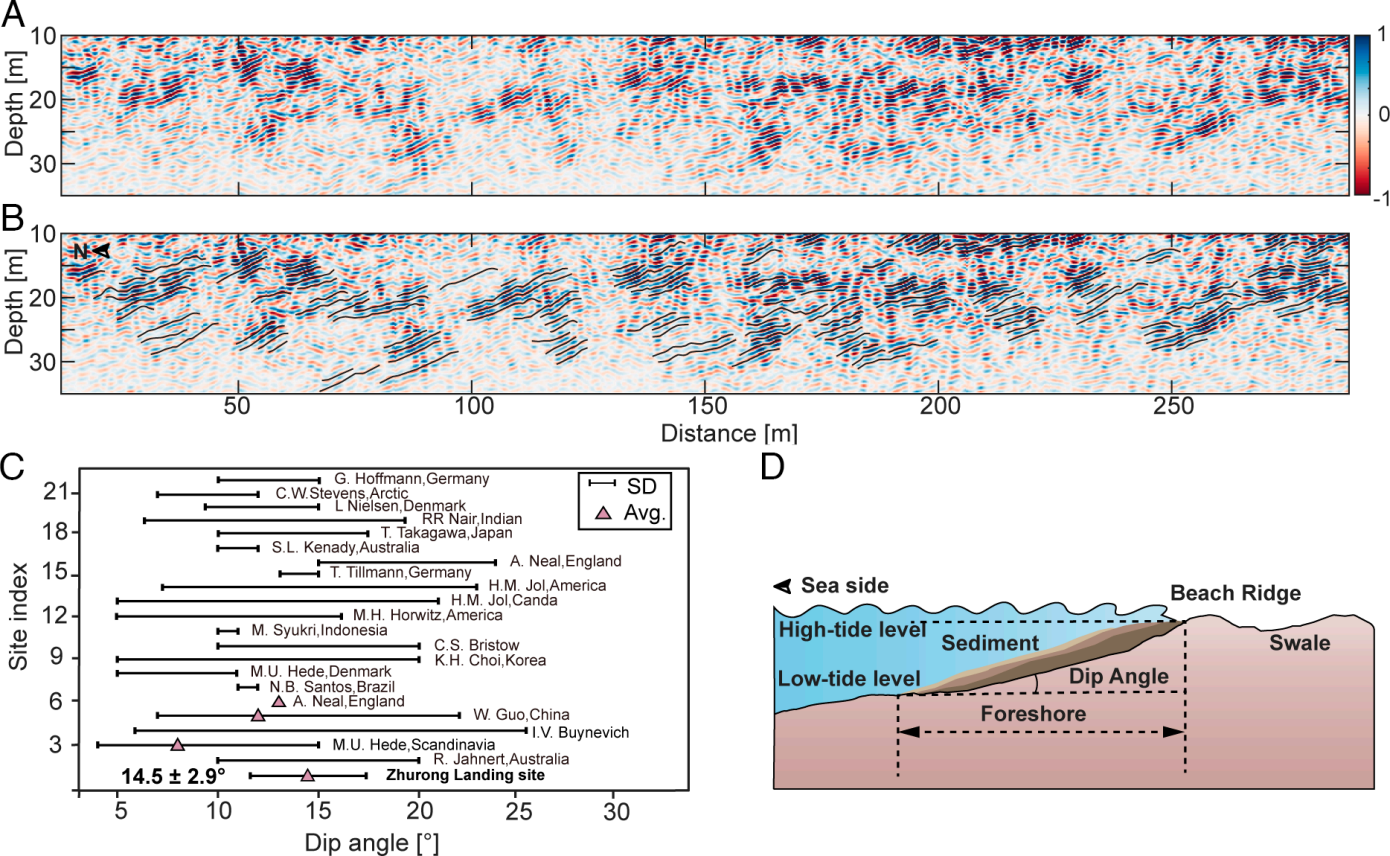
B-scans of the RoPeR low-frequency channel data revealing buried coastal sedimentary deposits. (*A* and *B*) Processed radargrams of RoPeR low-frequency channel from 12 to 288 m along the Zhurong rover traverse. The radar profile is limited to a depth of 10 to 35 m to highlight the dipping reflectors. Black lines in (*B*) denote the dipping reflectors identified as the coastal sedimentary deposits. The corresponding interpretive geologic cross-section at the same scale as (*A* and *B*) is shown in *SI Appendix,* Fig. S5. (*C*) Comparison of dip angles of coastal sedimentary deposits on the Earth and Mars. Some data are presented as mean values ± SD (28–48). The Zhurong landing site data are based on measurements from this study. (*D*) Sketch map illustrating the formation of multilayer dipping sedimentary deposits in a coastal environment.

The low-frequency channel data of RoPeR are preprocessed for self-test trace removal, trace-spacing regularization, background removal, and gain to refine observed structure (*Methods* and *SI Appendix*, Fig. S2). Hyperbola fitting constrains the dielectric permittivity distribution in the range of 3 to 7 (*SI Appendix*, Fig. S3). Kirchhoff migration then constrains time-to-depth conversions to recover true depths of observed subsurface structures. Then, we identify a total of 76 subsurface dipping reflectors at depths of 10 to 35 m. All of these reflectors dip toward the northern lowlands (Fig. 2 *A* and *B* and *SI Appendix*, Fig. S4). These reflectors are broadly uniformly distributed along the RoPeR survey traverse and dip at shallow inclinations from the southern highlands to the northern lowlands. These inclinations are normally distributed in the range of 6° to 20° with an average value of 14.5° and a SD of 2.9° (Fig. 2*C*).

Comparisons between the RoPeR results on Mars and geometries of terrestrial coastal deposits imaged by GPR present strikingly similar features (*SI Appendix*, Fig. S6). The dipping reflectors in southern Utopia Planitia and Bay of Bengal beach deposits both incline toward the lowland (ocean) direction and exhibit similar structures. Moreover, multiple dipping reflectors can be observed at the same position but at different depths in both the Martian and terrestrial radar results. These dipping multilayers are generally parallel to each other and are typical of sedimentary structures in coastal environments, formed by different deposition processes of varying tidal energies (28). Furthermore, we analyze the dip angles of marine progradation layers from a total of 21 different coastal areas on Earth, finding that the angles of dipping reflectors range from 4° to 26° (refs. 28–48). The dip inclinations from RoPeR in southern Utopia Planitia agree well with those of coastal sedimentary deposits on the Earth. We thus interpret the dipping reflectors in southern Utopia Planitia as coastal sedimentary deposits formed by ancient ocean waves and possibly composed of sand and pebble gravels transported by tidal currents.

## Discussion

Prior data from the radar images recorded by Mars’ Subsurface exploration (RIMFAX) onboard the Perseverance rover also detected dipping reflectors in Jezero Crater. These buried structures were interpreted as having either a magmatic or sedimentary origin (49). Maximum dips of the inclined layers in Jezero crater reach 15°, closely matching our results. The different interpretations of the RoPeR and RIMFAX profiles are due to the following (50): 1) relative permittivities from the RIMFAX data average ~9, significantly larger than the RoPeR estimate of ~3 to 7 (*SI Appendix*, Fig. S3); 2) the dipping reflectors in the RoPeR profiles are identified along most of the traverse (*SI Appendix*, Fig. S4), whereas the reflectors in the RIMFAX profiles are spatially discontinuous and compatible with magmatic layering in a differentiated magmatic body or document multiple aqueous episodes (figure 2 of ref. 50). These differences suggest different past depositional environments for southern Utopia Planitia and Jezero Crater.

**Fig. 3.**
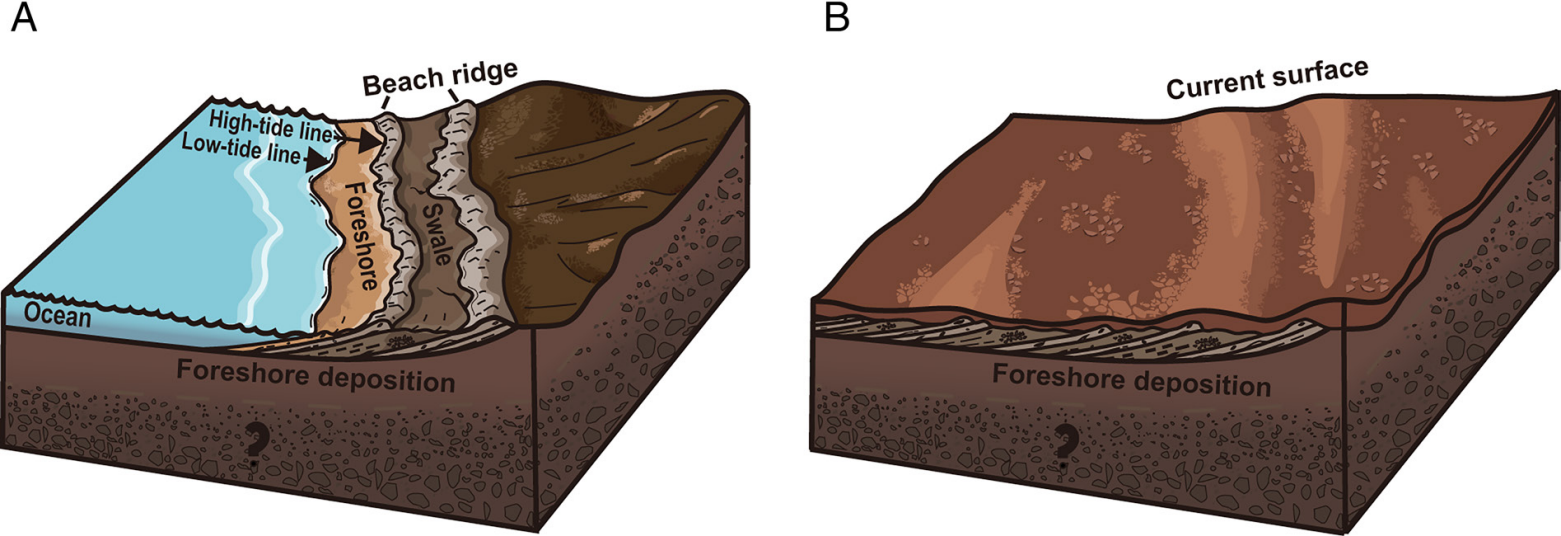
Schematic model of the formation process for tilted sedimentary terrain at the Zhurong landing Site. (*A*) Stratified structures formed under tidal sedimentation, (*B*) with the ancient shoreline regressed, liquid water disappeared and sedimentation ceased. Then long-term physical and chemical weathering altered the properties of the rocks and minerals, leading to the formation of a Martian surface layer. Consequently, the sedimentary deposits were covered by the current Martian surface soil.

Besides ocean sedimentary deposits, fluvial, igneous, and aeolian deposits can all form reflectors with dipping layers. However, the terrain around the Zhurong landing site is relatively flat and lacks typical surface or subsurface fluvial features of valley networks (51), disfavoring a fluvial origin. Previous studies from SHARAD have shown subsurface reflectors characteristic of lava flows in the Tharsis and Elysium-Utopia regions (52), although reflectors do not exhibit characteristics of multiple and parallel structures. More importantly, typical lava flow relative permittivities are ~9 and thus significantly higher than the RoPeR average of ~4.4 spanning the limited range of 3 to 7, potentially discounting a lava origin. Finally, for aeolian deposits, dip angles exhibit significant variations in steepness across the dune with additional cross-bedding characteristics. If the RoPeR data were imaging buried aeolian-dune cross sets, the reflectors should present varying inclination angles or display cross-bedded features on radar images (figure 6 of ref. 53)—these are not observed. Therefore, we conclude that the dip reflectors under the Zhurong landing site are most consistent with those formed in a coastal environment. Additionally, we observe some less prominent reflectors that dip in the southward direction and connect with northward-dipping reflectors (e.g., *SI Appendix*, Fig. S7), collectively forming a dome-like feature. We propose that this structure corresponds to beach ridges at the back of the swash zone (Figs. 2*D* and 3*A*) (54).

The distribution of dipping reflectors observed in the RoPeR profiles spans a traverse length of ~1.3 km nearly perpendicular to the proposed shorelines. This reveals a prolonged record of foreshore progradation rather than a single, frozen-in-place foreshore environment. Visual inspection of Zhurong rover surface rocks has interpreted them as sedimentary rocks deposited during the regression of the ancient ocean (7). The features we interpret as foreshore deposits are deeper than 10 m. The boulders hosting structures interpreted as oceanic sedimentary structures in Zhurong images could readily be excavated or ejected by meteorite impacts. Similarly, the derived average value of relative permittivity of ~4.4 agrees well with those of the sedimentary deposits which are composed of silt and fine-to-medium-grained sand particles accompanied by a minor fraction of pebbles (7, 55).

RoPeR profiles in southern Utopia Planitia reveal numerous buried dipping structures consistent with deposition in coastal environments, adding key information to the geological evolution. It is also valuable subsurface evidence of ancient ocean sedimentary deposits supporting the past existence of an ocean in the northern lowlands of Mars. The shoreline-perpendicular depositional length is >1.3 km, indicating that these sedimentary deposits were laid down in the presence of tides and within a persistent and stable ancient ocean. Furthermore, the presence of these sedimentary deposits suggests wind and wave-driven longshore transport supplied this reach of the shoreline with a steady net influx of sediment. This is inconsistent with a small, localized body of water in two ways. First, a small body of water would limit the fetch and thus the formation of sediment-transporting waves required for longshore transport. Second, there must also have been a primary sediment source, likely a river, sufficiently distant to have had its channel-filling deposits or floodplain not directly imaged in the GPR data. The presence of a distant river supplying sediment to this area is consistent with the inferred fine-to-medium sand grain size of the foreshore deposits and indicates that stable liquid water at the Martian surface was at least a regional and sustained phenomenon, rather than merely localized and short-lived melt. Sediment sourced by local, wave-driven bedrock erosion alone is inconsistent with a net influx of sediment and progradation. For bedrock erosion at some distance to have been the primary sediment source would require that the source area remained erosional while the shoreline along the rover transect remained net depositional. This is unlikely given the tendency of eroding bedrock shorelines to eventually reach a static equilibrium shape (56) and thus provide less sediment over time. Subsequently, the liquid water gradually disappeared from the Martian surface as the climate dried, and the sedimentary deposits were gradually covered by regolith (57–60). Although the timing and duration of formation and retreat of the ancient ocean require further constraints, our finding suggests a warm and wet period on Mars spanning tens of millions of years, estimated from deposition rates of ~10 to 40 cm per thousand years from analogous coastal sedimentary deposits on Earth (61).

## Methods

### Data Processing.

The low-frequency GPR channel of RoPeR onboard the Zhurong rover features a pair of monopole antennas (*SI Appendix*, Fig. S1) (62). This channel operates within a frequency range spanning 15 to 95 MHz, enabling an effective probing depth of 100 m. We process reflector arrivals prior to 1,500 ns time-of-flight to improve the signal-to-noise ratio (SNR) and image the subsurface structures. The corresponding data processing workflow consists of the following steps:

1) Self-test trace removal: The self-test traces of the RoPeR low-frequency channel are specifically used for checking the status of the RoPeR module. These traces are marked as “Self-Test” in the corresponding data label file and require removal.
2) Trace-spacing regularization: The trace interval of the RoPeR low-frequency data measurement was initially set to 50 cm. Notably, after 17 August 2021, the trace spacing for this channel has been uniformly adjusted from 50 to 25 cm. An interpolation is applied to equalize the trace spacing to 25 cm, benefiting all subsequent data processing.
3) Conventional GPR preprocessing: Data preprocessing, including direct current (DC) removal, zero-time correction, background removal, and band-pass filtering are applied to improve the SNR of the RoPeR data.
  • DC removal. The DC components of the recorded RoPeR data are removed by subtracting the mean value of each A-scan.
  • Bandpass filtering. A band-pass filter in the frequency domain, using a Hamming window, is applied to suppress the noise. The cutoff frequencies are set to be 15 MHz, 50 MHz, 70 MHz, and 95 MHz, respectively.
  • Zero-time correction. Time-zero for the low-frequency channel is 212.5 ns according to the system setting. Thus, the data prior to 212.5 ns are removed.
  • Background removal. A sliding window with a size of 30 traces is set to subtract the mean value of each segmented data.
  • Gain. An envelope gain curve is applied to compensate for energy attenuation.
4) Permittivity estimation: Numerous hyperbolic reflectors are manually identified and an interactive adaptation method is used to calculate the velocity of the EM waves and subsurface permittivity.
5) Migration: Kirchhoff migration is applied to preprocessed radar profiles with a relative permittivity value of 4.4 (*SI Appendix*, Fig. S3).
6) Topographic correction: The topographic variation along the survey traverse is calibrated using the relative elevation data recorded by the rover.

### Hyperbola Fitting.

In coastal environments, small protrusions of beach ridges and heterogeneities in the sediments can act as point scatterers, generating hyperbolic reflections in the GPR data. The curvature of these hyperbolic reflections can be analyzed using the hyperbola fitting method to determine the radar wave velocity and estimate the relative permittivity of the subsurface media (63–65). The relative permittivity is vital in the accurate interpretation of subsurface features, image reconstruction by migration processing and time-to-depth conversion, attempted here. The subsurface relative permittivity can be estimated as

[1] $$\varepsilon_{r}=\frac{c^{2}(t-t_{0}{)}^{2}}{4((x-x_{0}{)}^{2}+h_{0}^{2})},$$

where *c* is the electromagnetic wave speed in a vacuum, with *x* and *t* representing the horizontal location of a point on the hyperbola and its corresponding two-way travel time in the subsurface, respectively. *t*_0_ is the two-way travel time of the electromagnetic wave in the atmosphere layer, accounting for the antenna height above the ground (*h* = 903 mm), as shown in *SI Appendix*, Fig. S1. The relative permittivity *ε*_r_ and depth *h*_0_ of the subsurface targets are determined by identifying the positions of the measurement points (*x*, *t*) on the hyperbolas in the radargrams.

### Kirchhoff Migration.

Kirchhoff migration is used to collapse diffraction hyperbolas and focus energy at reflector points, thereby enhancing the resolution and accuracy of subsurface imaging (66, 67). It utilizes Huygens’ Principle by treating each point on a wavefront as a secondary spherical wavelet source to enhance subsurface imaging. Its mathematical representation is

[2] $$P(x,z)=\frac{1}{2\pi }\sum_{i=1}^{n}\frac{\cos\theta }{\sqrt{\mathrm{vR}}}\frac{\partial }{\partial t}E_{i}(t_{i}(x,z)),$$

where *P*(*x*, *z*) is the wave field at position (*x*, *z*), *n* is the number of receiving points, *θ* is the reflector angle, *v* is the propagation velocity of the electromagnetic radar waves in the subsurface medium, *R* and *t_i_* are the two-way travel path and travel-time from transmitter to reflector and back to receiver, respectively, and *E_i_*(*t_i_*(*x*, *z*)) is the amplitude of the GPR signal recorded at the receiving point *i* at time *t_i_*.

## Supplementary Material

Appendix 01 (PDF)

## Data Availability

The Mars Rover Penetrating Radar level 2B datasets have been deposited in the Mars-Scientific Data Node of the Lunar and Planetary Data Release System, hosted by the National Astronomical Observatories of China (68). All other data are included in the manuscript and/or *SI Appendix*.
