# Supplementary material for: Ancient ocean coastal deposits imaged on Mars
Source: Proc Natl Acad Sci U S A. 2025 Feb 24;122(9):e2422213122. doi: 10.1073/pnas.2422213122 (PMC11892591; doi:10.1073/pnas.2422213122)
Supplement: Supplementary file 1 — Appendix 01 (PDF) [file pnas.2422213122.sapp.pdf]

PNAS

Supporting Information for
Ancient ocean coastal deposits imaged on Mars

Jianhui Li[a,1], Hai Liu[a,1,*], Xu Meng[a], Diwen Duan[a], Haijing Lu[a], Jinhai Zhang[b],Fengshou Zhang[c], Derek Elsworth[d,e], Benjamin T. Cardenas[e], Michael Manga[f,*], Bin Zhou[g], Guangyou Fang[g,*]

*Corresponding authors: Hai Liu, Michael Manga, Guangyou Fang
Email: hliu@gzhu.edu.cn; mmanga@berkeley.edu; gyfang@mail.ie.ac.cn

This PDF file includes:

Figures S1 to S7
SI Reference

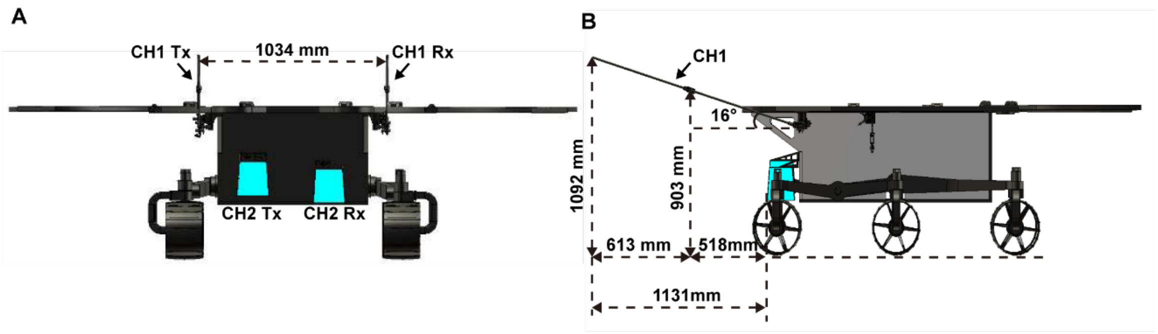

Fig. S1. Illustration of RoPeR. (*A*) The front view and (*B*) the side view.

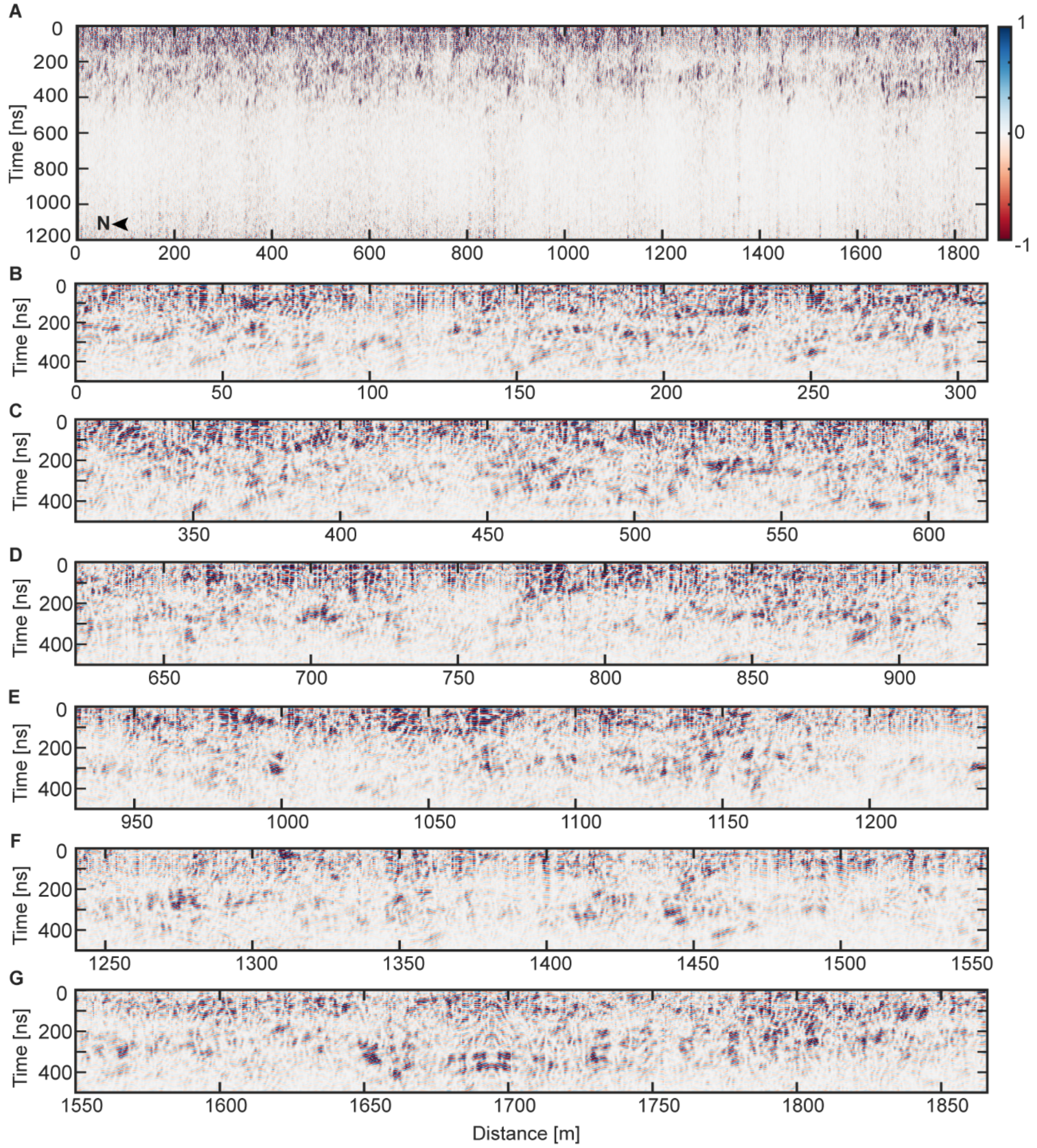

Fig. S2. Preprocessed radar profiles before migration. (*A*) Complete radar profile of RoPeR CH1, spanning the entire survey range (0–1860 m). Local radar profiles below the surface, ranging from (*B*) 0-310 m, (*C*) 310-620 m, (*D*) 620-930 m, (*E*) 930-1240 m, (*F*)1240-1550 m, and (*G*) 1550-1860 m, respectively.

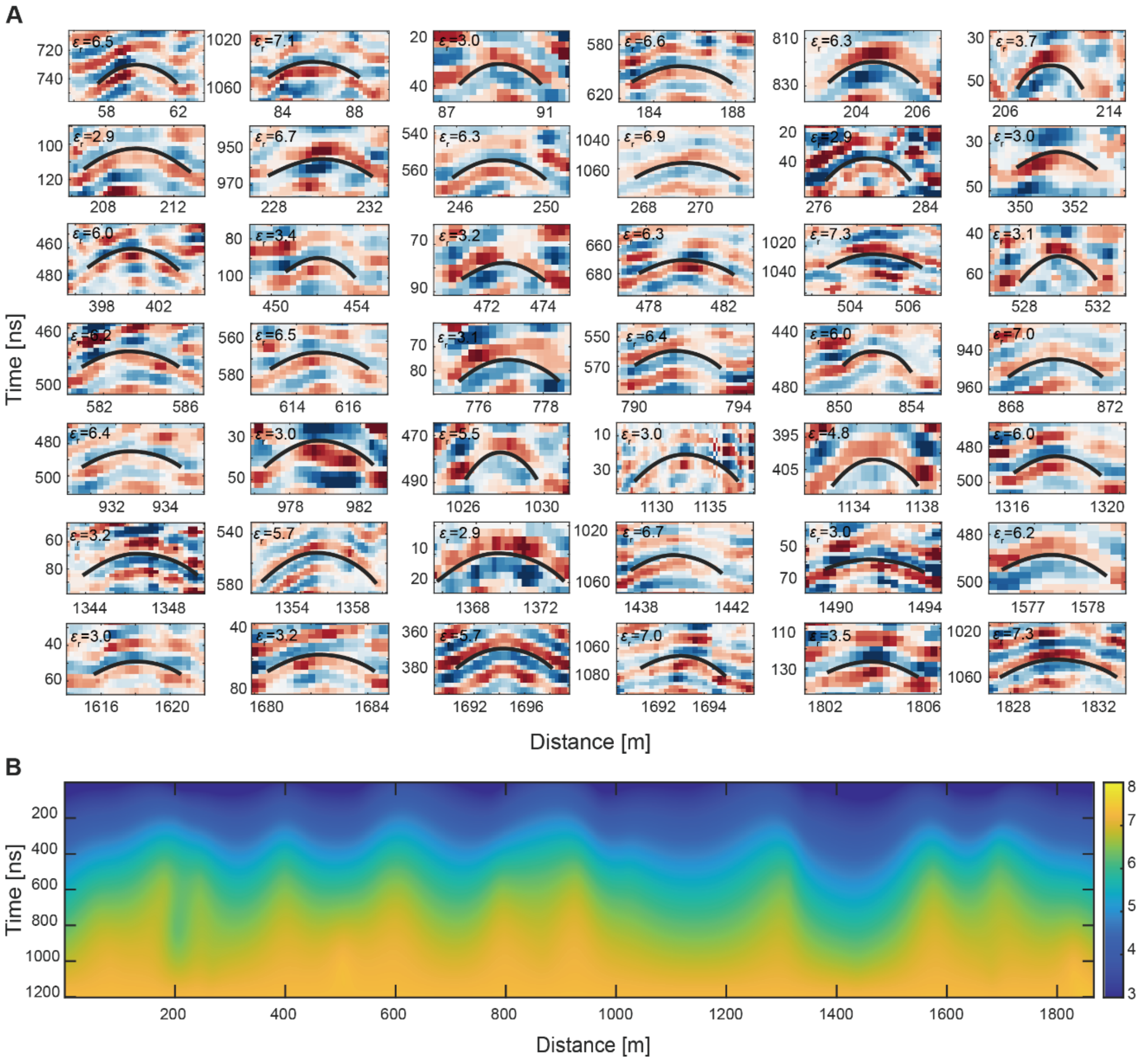

Fig. S3. Permittivity estimation results by the hyperbola fitting method. (*A*) Examples of the selected hyperbolas used for the permittivity estimation. The colorbars are adjusted individually for better visualization of each hyperbola. (*B*) Subsurface distribution of permittivity along the survey line of the Zhurong rover. Note that the dipping reflectors identified in RoPeR low frequency channel data are distributed before 500 ns, and the average relative permittivity for this range is 4.4.

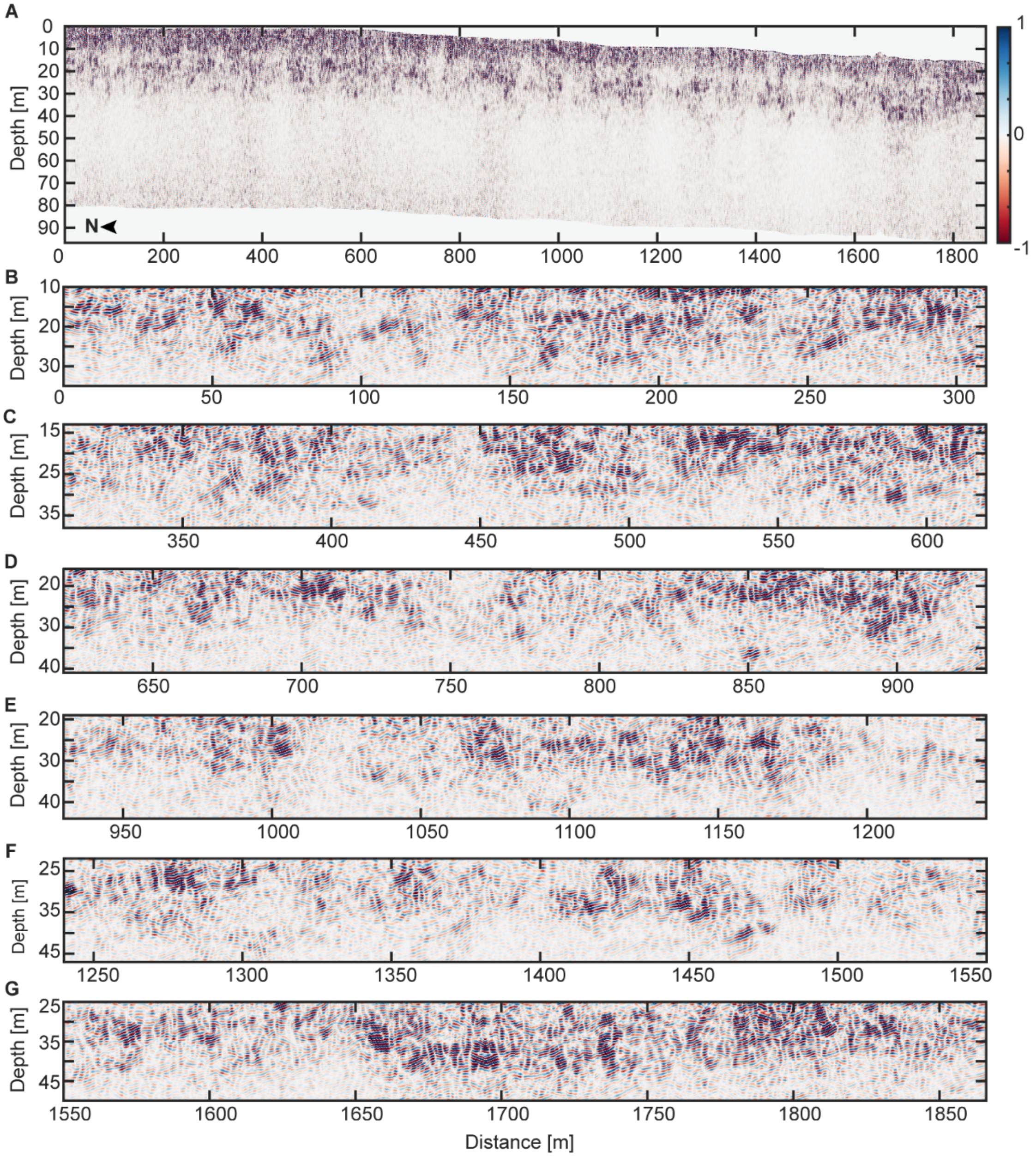

Fig. S4. Migrated radar profiles of the RoPeR CH1 along the survey line of the Zhurong rover. (*A*) Migrated radar profile of RoPeR CH1 with a terrain sloping to the north. Local radar profiles below the surface, ranging from (*B*) 0-310 m, (*C*) 310-620 m, (*D*) 620-930 m, (*E*) 930-1240 m, (*F*)1240-1550 m, and (*G*) 1550-1860 m respectively. Note that the average relative permittivity of 4.4 before 500 ns is used for Kirchhoff migration to ensure the accurate height estimation of the dipping reflectors.

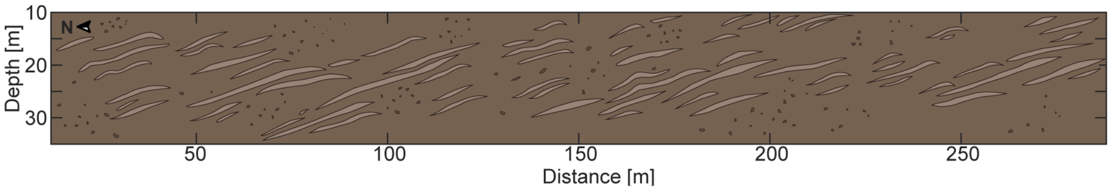

Fig. S5. Geologic interpretation of subsurface features derived from the processed radargrams shown in Fig. 2*A* and *B*, presented at the same scale.

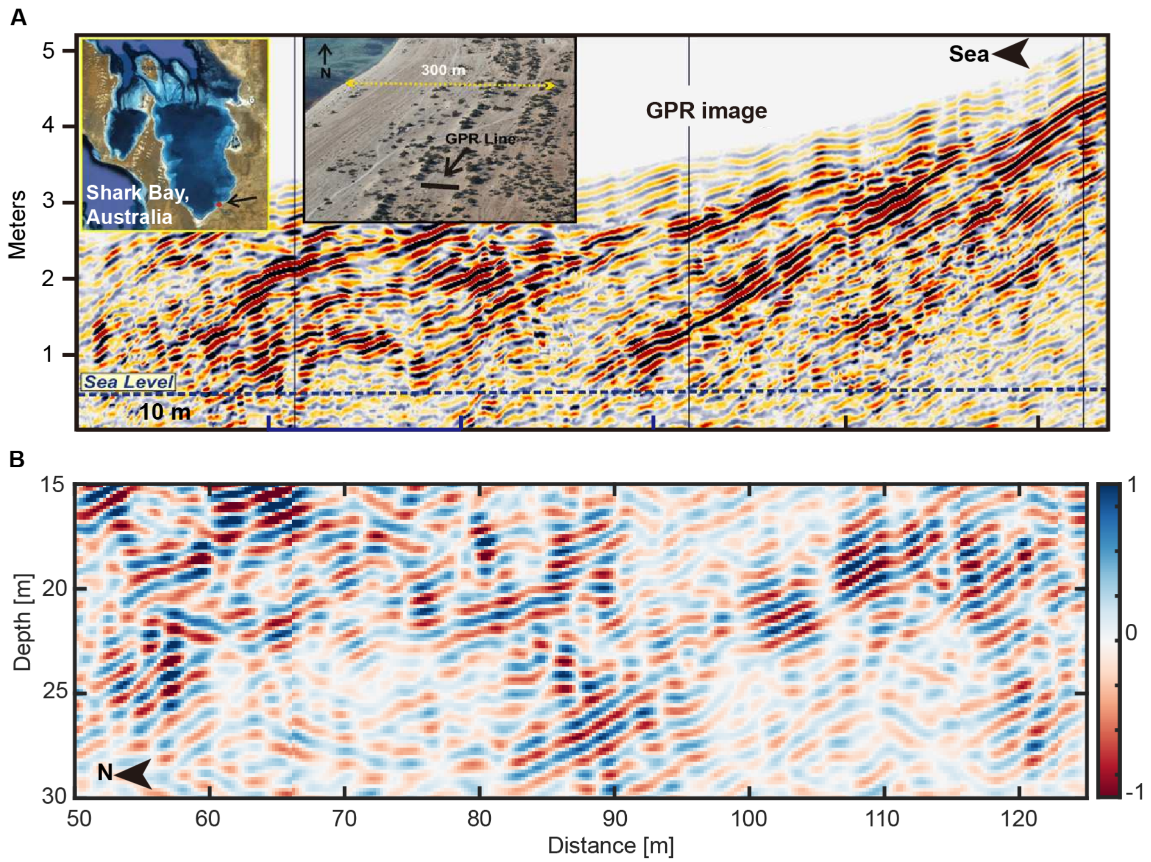

Fig. S6. Comparison between the dipping reflectors detected on Mars with those of marine sedimentary deposits on Earth. (*A*) The GPR radargrams detected in Shark Bay, Australia (1). Note that the dipping reflectors represent marine sediments formed in the foreshore area, (*B*) The processed radar profile of RoPeR low-frequency channel. The dipping reflectors show similar features to those on Earth.

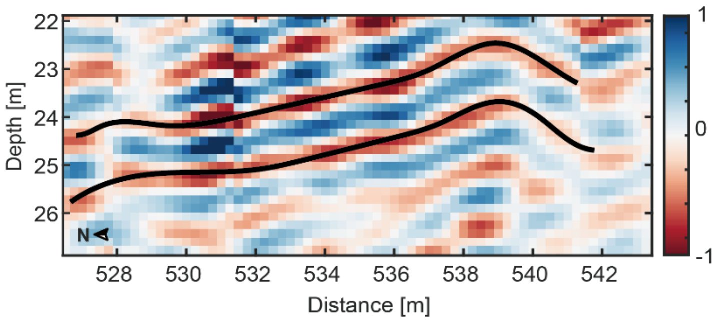

Fig. S7. B-scans of the RoPeR low-frequency channel data revealing a potential beach ridge, which is marked by Black curves.

SI Reference

1. R. Jahnert, O. De Paula, L. Collins, E. Strobach, R. Pevzner, Evolution of a coquina barrier in Shark Bay, Australia by GPR imaging: architecture of a Holocene reservoir analog. *Sediment. Geol.* **281**, 59–74 (2012).
